# Combined Effects of Spirulina Liquid Extract and Endurance Training on Aerobic Performance and Muscle Metabolism Adaptation in Wistar Rats

**DOI:** 10.3390/nu17020283

**Published:** 2025-01-14

**Authors:** Jordi Vignaud, Céline Loiseau, Martine Côme, Isabelle Martin, Rova Rasoanarivo, Josiane Hérault, Claire Mayer, Olivier Lépine, Lionel Ulmann

**Affiliations:** 1 BiOSSE, Biology of Organisms, Stress, Health, Environment, Institut Universitaire de Technologie, Département Génie Biologique, Le Mans Université, 53020 Laval, France; jordi.vignaud@univ-lemans.fr (J.V.); celine.loiseau@univ-lemans.fr (C.L.); martine.come@univ-lemans.fr (M.C.); isabelle.martin@univ-lemans.fr (I.M.); rova.rasoanarivo@gmail.com (R.R.); josiane.herault@univ-lemans.fr (J.H.); claire.mayer@univ-lemans.fr (C.M.); 2 AlgoSource, 44350 Guérande, France; olivier.lepine@algosource.com

**Keywords:** endurance training, *Spirulina* liquid extract, muscle performance, antioxidants

## Abstract

Background: Physical activity, such as running, protects against cardiovascular disease and obesity but can induce oxidative stress. Athletes often consume antioxidants to counteract the overproduction of reactive oxygen and nitrogen species during exercise. *Spirulina*, particularly its phycocyanin content, activates the Nrf2 pathway, stimulating antioxidant responses. Studies show that phycocyanin enhances antioxidant defenses and reduces inflammation, potentially improving muscle adaptation and recovery. This study evaluates a *Spirulina* liquid extract (SLE) supplementation during endurance training, hypothesizing that phycocyanin improves oxidant status and performance in soleus and extensor digitorum longus muscles. Methods: Three-week-old male Wistar rats were divided into four groups: a sedentary control group (C), a sedentary group supplemented with SLE (SP), an endurance training group (T), and an endurance training group supplemented with SLE (SPT). After 8 weeks of treadmill training, blood and muscle were collected. Biochemical parameters and gene expression analyses were performed to assess the effects of training and supplementation. Results: The maximal aerobic speed improved significantly in the SPT group. Plasma lipid profiles showed a reduction in triglyceridemia, cholesterolemia, and atherogenic index in the trained groups, especially with SLE supplementation. Muscle malondialdehyde levels decreased in the SPT group compared to T. Gene expression analysis revealed upregulation of Nrf2 and mitochondrial biogenesis genes in both muscles, with differences between groups for genes related to glycogen storage and β-oxidation. Conclusions: This study demonstrated that SLE supplementation enhanced exercise performance and promoted muscle molecular adaptations. These findings suggest SLE as a promising functional food supplement for athletes, optimizing recovery and performance.

## 1. Introduction

Physical activity is recognized as an essential element of a healthy lifestyle that protects against cardiovascular disease and obesity [1,2]. Running is the most accessible activity and is practiced worldwide for a healthy life, for competition, or for weight loss. Acute exercises can lead to elevated stress levels, such as oxidative stress or inflammation. When repeated, they are considered as endurance training sessions where adaptations are able to improve mitochondrial biogenesis and antioxidant status [3,4]. To improve these effects and to increase training volume, athletes consume various food supplements such as proteins, carbohydrates, omega-3 polyunsaturated fatty acids (PUFA), minerals, vitamins, or antioxidants [5]. Antioxidants in athletes’ diets help compensate for the overproduction of reactive oxygen and nitrogen species (RONS) and inflammation during aerobic exercise [6]. These supplements aim to improve performance, muscle adaptation, or recovery, and further enhance the benefits of exercise [7]. During endurance training, consistent ATP production is essential, requires the high activation of several pathways, and increases mitochondrial activity. At the same time, the activity of oxidase xanthine and NADPH induces a high production of RONS which can lead to oxidant stress [8]. These RONS can affect performance, specifically during long endurance events, exhaustive exercise, or extended and repeated high-intensity exercise bouts [9,10]. This is explained by the impact of RONS on DNA damage, protein breakdown, lipid peroxidation, and apoptosis, which leads to impaired skeletal muscle excitation–contraction coupling [11,12,13]. In contrast, other studies have demonstrated that some RONS production is necessary for muscle and mitochondria adaptations after exercise [14,15,16]. These adaptations occur through specific muscle genes, such as nuclear respiratory factor 1 (Nrf1), or mitochondrial transcription factor A, which are activated by peroxisome proliferator-activated receptor gamma coactivator 1-alpha (PGC1α) after being stimulated by RONS [14,17]. Then, primary antioxidant supplementation, such as vitamin C or E, can directly scavenge and neutralize the RONS. However, this type of supplementation requires caution due to hindering muscle adaptation in both resistance and aerobic training conditions. [9,15,18,19]. Conversely, other studies suggest that secondary antioxidants, which activate the nuclear factor-like 2 (Nrf2) pathway, can stimulate antioxidant response elements (ARE), potentially enhancing muscle performance and recovery [20,21,22].

Among food supplements that can provide bioactive molecules such as proteins, carbohydrates, omega-3 PUFA, minerals, vitamins, or antioxidants, microalgae are promising sources [23]. In the various world of microalgae, *Arthrospira platensis*, or *Spirulina*, is identified as cyanobacteria and is known to have antioxidant activity due to its high content in the C-phycocyanin (PC), a water-soluble protein [24]. This protein is composed of α and β subunits and is bound to phycocyanobilin [25]. PC is recognized as a Nrf2 activator that regulates genes for the ARE response [26]. Studies have demonstrated the effects of PC with a strong antioxidant effect through Nrf2 pathways [27] and anti-inflammatory activity [28]. *Spirulina* liquid extract (SLE) obtained from *Arthrospira platensis* is a liquid extract concentrated in phycocyanin produced by AlgoSource (Guérande, France). In recent studies, SLE has been reported to increase antioxidant defense in mice and hamster models after hypercaloric diet consumption [29,30]. Another study demonstrated in Sprague-Dawley rats that PC alone could increase ARE without changing the content of thiobarbituric acid reactive substances (TBARS) in the gastrocnemius muscle, a biomarker of lipid peroxidation [31]. This suggests that the mechanism of action of PC might not be directed against RONS, but rather stimulate the activation of AREs, which are themselves regulated by a Nrf2 transcription factor.

This study aims to evaluate the effects of the SLE food supplement, in combination with endurance training, on biochemical parameters and gene expression related to lipid metabolism, glucose metabolism, oxidative status, and mitochondrial pathways in the soleus (SOL) and extensor digitorum longus (EDL) muscles. We propose that the antioxidant activity of PC contained in SLE could act on the Nrf2 transcription factor and improve oxidant status. This could lead to an improvement in performance measured by maximal aerobic speed (MAS) and molecular adaptation in two muscles with different fiber types.

## 2. Materials and Methods

### 2.1. Animals, Supplementation, and Diets

Thirty-two 3-week-old male Wistar rats, with an initial weight of 120 ± 10 g, were obtained from Janvier Labs (Le Genest-Saint-Isle, France). They were housed four per cage under controlled conditions of temperature (22 ± 2 °C), humidity (40–60%), and with a 12 h light/dark cycle. During a week of acclimatization, all animals were fed ad libitum with the standard diet A04 (SAFE, Augy, France) and had free access to tap water.

After a week of adaptation, the animals fed with the standard diet A04 (SAFE, Augy, France) were randomly divided into four groups of eight rats according to their running capacity and food supplement associated with diet. The following groups were determined: a sedentary control group (C); an endurance training group (T); a sedentary group with a diet supplemented with SLE (SP); and an endurance training group with a diet supplemented with SLE (SPT). According to the manufacturer’s information, the standard diet A04 is composed of carbohydrates (72%), proteins (19%), and lipids (8%) for a total energy of 3.35 kcal/g. The SLE was added to drinking water at a concentration of 1.1 mg/100 g of bodyweight. The SLE (Spirulysat^®^, number of patent: 17 52452) was kindly supplied by AlgoSource (Guérande, France). Its composition (mg/100 mL extract) is as follows [29]: phycocyanin (mg/L) 883; carbohydrates (g/100 g) 0.4; amino acids (g/100 g); threonine 0.009; aspartate 0.0187; alanine 0.0168; arginine 0.0138; leucine 0.0164; glutamate 0.022; ions (mg/kg): Fe^2+^ 1.05, Mg^2+^ 18, Ca^2+^ 330, K^+^ 55, Cu^2+^ 0.2. According to its composition (the calorie value is 7 kJ/100 g dry matter) and the daily provided dose, the SLE caloric supplement was considered negligible. The food supplement was provided for 8 weeks. The daily food and water consumption were evaluated for 8 weeks. The body weight gain of the rats was monitored three times a week and before training.

All thirty-two rats were euthanized at the end of the experimental protocol without any sign of pain, suffering, or distress. According to the animal well-being respect, no exclusion criteria were used. Similarly, no inclusion criteria were necessary. The experimental procedures, the justification of the use of an animal model, and the details related to the respect of the rule of 3R (housing and husbandry, well-being, animal care, and monitoring) were approved by the Ethical Committee 006 Pays de la Loire and by the French Ministry of National Education, Higher Education and Research (procedure APAFIS #40394-202301111324826 v6, 23 February 2023).

### 2.2. Endurance Training Protocol

During one week, all groups were adapted to a treadmill (BIOSEB, Vitrolles, France), based on gradually increasing speed from 11 to 21 cm/s (Table 1). This week of acclimatization was planned to limit any stress phenomena related to the equipment that will be used and to determine the running animals. Moreover, the established limit points and observations made during the running protocols helped to avoid the installation of stress.

Rats with running dispositions were selected, and groups were formed based on the initial weighing and according to food supplement (T and SPT). For these groups, on the last day of the adaptation week, maximal aerobic speed (MAS) was measured to adjust the treadmill speed for the first week of training (Table 1). To force the rats to run, each running track was equipped with a grid at which short-term and low-intensity electrical stimulation could be administered. The number of stimulations could vary from 30 to 90 according to the race time. Indeed, according to our previous observations in the laboratory, it was accepted that rats could be stimulated once per minute. To limit any stress or anxiety phenomenon, a grid for evaluating the response of the rats to this learning was set up. The treadmill was able to count the number of stimulations for each rat. Moreover, the state of fatigue of each rat was quantified visually by the experimenter present throughout the race. Thus, when the animal remained on the grid for more than 3 s, it was taken out and put back with its congeners until the next day. The training sessions were conducted using a treadmill and were 5 days out of 7. The MAS protocol began with a running speed of 15 cm/s, increasing by 3 cm/s every minute, then by 2 cm/s in the following minute, and alternating between 3 cm/s and 2 cm/s increments until exhaustion (adapted form of [32,33]). The MAS was measured before each training block, allowing the training speed of the following block to be adjusted (Table 1). Indeed, endurance develops when the running speed corresponds to 60–80% of the MAS. Therefore, the first block of 3 weeks was structured as follows: week 1, 60% of the MAS; week 2, 70% of the MAS; week 3, 80% of the MAS. Starting from block 2, sprints were added in addition to the training speed to improve the MAS and further stress the mitochondrial tissue [34]. Additionally, a short warm-up of 6 min at 15 cm/s up to 30 cm/s was applied before each MAS test or daily training session [35].

### 2.3. Glucose Tolerance Test

At the beginning of the 7th week, a glucose tolerance test (GTT) was performed on all animals. The training activity was stopped two days before to avoid any training effects, and the rats were fasted the day before the test. Briefly, each rat was given an intraperitoneal injection of glucose (1 g/kg BW) followed by measurement of blood glucose level from the tail vein with a glucometer (Freestyle Papillon Vision, Abbott Diabetes Care, Rungis, France). The volume of insulin solution used for intraperitoneal injection complied with the recommendations. Specifically, the volume was less than 1 mL per 100 g of bodyweight. Concerning animal welfare, a 20% glucose solution was ready to be injected in case of hypoglycemic attack (glycemia < 0.7 g/L) or absence of reaction. The glucose levels were measured at 0 (before the glucose challenge), 15, 30, 60, 90, and 120 min after injection of glucose. On the test day, no exercise was performed, and running was resumed the following day.

### 2.4. Blood and Muscle Sampling

Following the last day of training, 3 days were allowed to avoid any acute effects of exercise [36,37,38]. The rats were anesthetized with a diazepam (5 mg/mL) and ketamine (Imalgene 1000, Boehringer Ingelheim Animal Health, Lyon, France, 100 mg/mL) (4:3, *v*/*v*) mixture administered intraperitoneally, before exsanguination. All the blood was collected by abdominal aortic puncture. Restraint and injection during anesthesia were performed by qualified technicians and researchers in animal experimentation, which avoided any stress on the animal. Moreover, anesthesia was performed in a room isolated from other congeners. The chemical molecules and doses administered were adapted to the animal model used. A reflex test was performed to check the state of anesthesia and a visual check of the death of the animal was performed after total exsanguination.

Blood was collected via the abdominal aorta, with an anticoagulant ACD (citric acid, citrate, dextrose) solution composed of 130 mM citric acid, 170 mM trisodium citrate, and 4% dextrose added to the collection tube. The blood samples were then centrifuged at 1000× *g* for 10 min. The supernatant corresponding to plasma and the red blood cell pellet were collected, aliquoted, and stored at −80 °C.

The right leg soleus (SOL) and extensor digitorum longus (EDL) muscles were taken and aliquoted for biochemical and molecular biology analyses. All samples were stored at −80 °C. Additionally, tibial length (TL) was measured using a caliper graduated in centimeters. All collected tissues were normalized to 100 g of body weight, expressed as g/100 g BW, as well as to tibial length, expressed as g/cm TL.

### 2.5. Plasma Parameter Analysis

The plasma concentrations of triglycerides (CAK-1086 Clinisciences, Nanterre, France), total cholesterol (KA3709 Clinisciences, Nanterre, France), high-density lipoprotein cholesterol (90406 Biolabo, Maizy, France), insulin (17475383 Thermo Fisher Scientific, Illkirch-Graffenstaden, France), and interleukin-6 (Ab100772 Abcam, Cambridge, UK) were measured according to the manufacturer’s instructions. The no high-density lipoprotein cholesterol (non-HDL-Chol) was calculated with total cholesterol minus HDL-Chol [39]. The atherogenic index of plasma (AIP) was determined with the following formula: $AIP={log}_{10}(\frac{TG}{HDL-Chol})$ [40].

### 2.6. Muscle Malondialdehyde Analyses

Muscle tissue (~30 mg) was homogenized in 300 µL of PBS (pH 7.4) on ice to prevent lipid degradation. The homogenate was vortexed and centrifuged at 12,000× *g* for 10 min at 4 °C. The supernatant was collected, and 150 µL was mixed with an equal volume of 1% thiobarbituric acid (TBA, prepared with 50 mM NaOH and heated to 100 °C until clear). An equal volume of 25% HCl was then added and vortexed again. The mixture was incubated in a 100 °C water bath for 10 min, followed by a 5-min incubation on ice. After cooling, 225 µL of n-butanol was added, vortexed, and centrifuged at 12,000× *g* for 10 min. The upper phase was measured at 532 nm. This method was adapted from Winterbourn et al. [41].

Muscle sample total protein was quantified using the Bradford method and malondialdehyde (MDA) concentration was normalized to total protein concentration.

### 2.7. Muscle RNA Extraction and Gene Expression

Thirty mg of each muscle was used for total RNA extraction, using the RNeasy Fibrous Tissue Mini Kit (Abcam, ID: 74704, Cambridge, UK). The RNA was quantified by measuring its absorbance at 260 nm using the Nanodrop One (Thermo Fisher Scientific, Illkirch-Graffenstaden, France).

Reverse transcription of the RNA extracts was conducted using a M-MLV Reverse Transcriptase (RT) (Promega) in a GeneAmp PCR System 9700 (Thermo Fisher Scientific, Illkirch-Graffenstaden, France). In the first step, 1 μg of RNA of each sample, 500 ng of oligo(dT)15 primers, and 500 ng of random hexamer primers was incubated in a volume of 15 μL at 70 °C for 5 min for annealing. Then, in a second step, 10 μL of a mix of dNTP (0.5 mM), M-MLV RT (200 U), RNAse inhibitor (25 U), and M-MLV 1X buffer was added and the tubes were incubated at 42 °C for 1 h, and then at 70 °C for 5 min, to inactivate the enzyme.

The produced cDNA was diluted four times for SOL and eight times for EDL before use. Quantitative PCR was performed in a QuantStudio 3 qPCR system (Applied Biosystems) with a standard program of an initiation step at 95 °C for 2 min followed by 40 amplification cycles alternating 95 °C for 15 s for annealing, and 60 °C for 1 min for elongation. A volume of 5 μL of cDNA was added to a 15 μL mix containing 10 μL of GoTaq qPCR Master mix 2X (Promega, Madison, WI, USA), 1 μL of each primer forward and reverse (10 μM), and 3 μL of nuclease-free water.

The primers were designed using Primer3 software 4.1.0 with the following parameters: an annealing temperature (Tm) of 60 ± 2 °C, amplification product lengths between 150 and 200 base pairs, primer lengths of 20 ± 3 base pairs, complementarity scores near 0, and primer pairs positioned as close as possible to the 3′ end of the gene sequence. A detailed list of all primers is provided in Table 2. To calculate relative expression, tubulin α (Tubα) was chosen as the housekeeping gene. Finally, the expression level was determined by the 2^(−ΔΔCt)^ method and normalized to tubulin α as the housekeeping gene [42].

### 2.8. Statistical Analysis

The statistical power analysis was performed using G*Power (version 3.1). Since the study involved independent groups without repeated measures, the “F test” family was selected, and the tool “ANOVA: Fixed effects, omnibus, one-way” was used. The margin of error in the power analysis was set at 5% (α = 0.05), and the desired power value was 95% (1 − β = 0.95). The calculation was performed using the mean values obtained in this study to estimate the effect size. Based on these calculations, we determined that a sample size of **n = 5 per group** was sufficient for statistical validity. However, flexibility was allowed for sample sizes between **n = 5–8 per group** to account for potential exclusions due to aberrant values or measurement failures. In addition, the exclusion of a value was verified by a ROUT statistical test (Q = 1%), and whether the value was twice different from the average of the other values. Statistical analyses were generated with Prism (GraphPad Software Inc., San Diego, CA, USA). Descriptive measurements were presented as mean ± SEM (standard error of the mean). The normality was verified with Shapiro–Wilk or Kolmogorov–Smirnov tests. The significance level was set at *p* < 0.05. Differences between groups were assessed using a one-way ANOVA with parametric tests (Brown–Forsythe test and Bartlett’s test) or non-parametric tests (Kruskal–Wallis test), depending on normality. Post-hoc comparisons were performed using Tukey’s or Dunn’s tests. For paired data, the Holm–Sidak’s multiple comparisons test was used. When comparing two independent groups, a student’s *t*-test was performed with a normal distribution. If the data did not follow a normal distribution, a Mann–Whitney U test was conducted.

## 3. Results

### 3.1. Nutritional Monitoring

**Figure 1:**
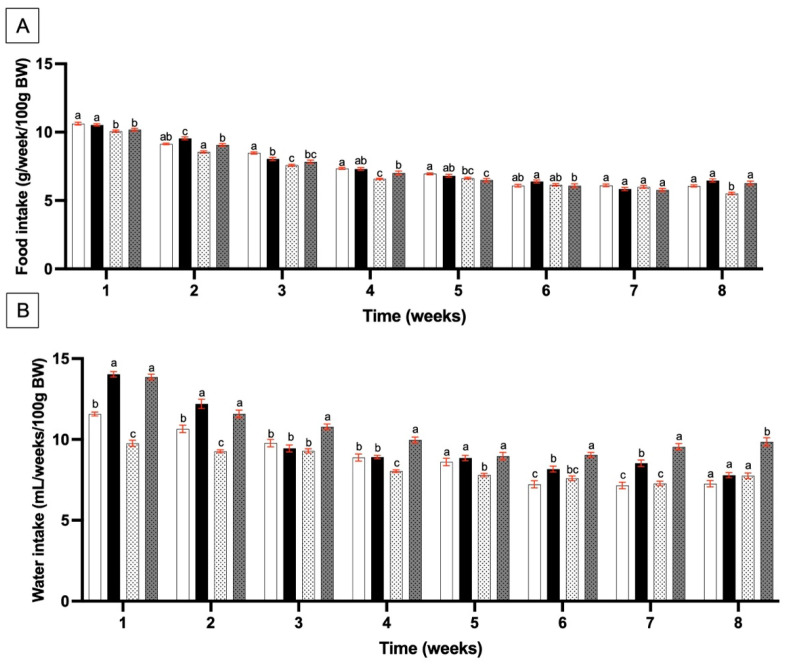
Nutritional monitoring. The figure shows the average food (**A**) and water intakes (**B**) relative to 100 g of bodyweight (g/week/100 g BW) in rats over 8 weeks. C (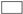), sedentary control group; SP (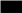), sedentary group supplemented with SLE; T (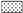), endurance training group; and SPT (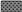), endurance training group supplemented with SLE. Each bar represents the mean ± SEM (n = 8) for each group. Significant differences between groups at each time point are indicated by different letters (a > b > c) above the bars, with *p* < 0.05 considered statistically significant.

Food and water intake were measured daily, with SLE being added to the water for the SP and SPT groups. During the training protocol, food intake (Figure 1A) significantly decreased in the T group compared to the C group between weeks 1 and 4.

From the 5th week onwards, stagnation appeared in all groups; although, in the last week, food intake in the T group was significantly lower compared to all other groups. No major effect of SLE supplementation was observed, though a significant increase in food intake was noted at weeks 2, 4, and 8 in the SPT group compared to the T group. The mean food intake over the 8 weeks did not show any significant differences between the different groups (Table 3).

### 3.2. Bodyweight Evolution

**Figure 2:**
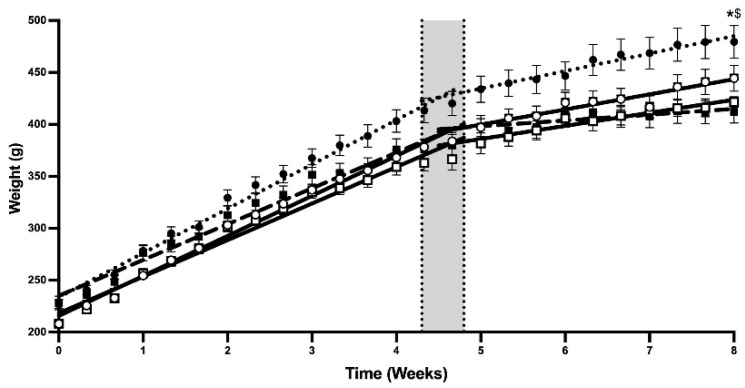
Bodyweight evolution. The figure shows bodyweight evolution of rats over 8 weeks. C (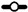), sedentary control group; SP (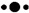), sedentary group supplemented with SLE; T (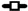), endurance training group; and SPT (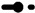), endurance training group supplemented with SLE. Each point represents the mean ± SEM (n = 8) for each group. * indicates a difference between C and T, and $ indicates a difference between SP and SPT for *p* < 0.05.

Bodyweight (BW) was measured three times per week over the 8-week period. A continuous increase in BW was observed in all groups until the 4th week (Figure 2).

Similar to food and water intake, a decrease in the BW evolution was observed after the 5th week. At the end of the 8th week, a decrease in BW was observed in the T and SPT groups compared to the C and SP groups, with no significant effect of SLE (Figure 2). Pearson correlations were calculated between food intake and weight gain (Table 3). No significant correlation was found before or after the 5th week, or at the end of supplementation.

### 3.3. Muscle Weights and Tibia Length

After euthanasia, collected muscles were weighed, and tibia length was measured. The SOL and EDL muscles, which are engaged during running, were activated during endurance training on the treadmill. When the SOL muscle mass was normalized to BW, a significant increase in mass was observed in the T group compared to the C group (Table 3). However, no such difference was observed with SLE supplementation. In contrast, for the EDL muscle, a significant increase in mass was observed in the SP and SPT groups, but not in the C and T groups. When muscle mass was normalized by tibia length, no differences were observed between the groups (Table 3).

### 3.4. Glucose Tolerance Test

**Figure 3:**
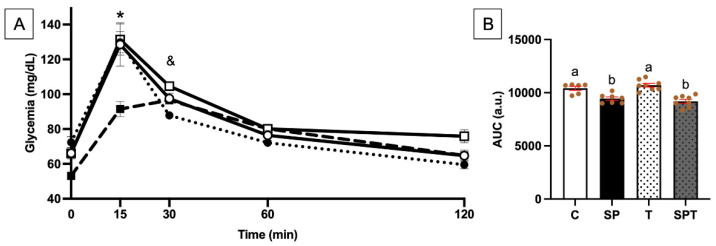
**Glucose Tolerance Test (GTT).** (**A**): the figure represents glycemia response (mg/dL) over 120 min following glucose administration in different groups; C (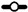), sedentary control group; SP (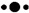), sedentary group supplemented with SLE; T (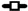), endurance training group; and SPT (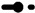), endurance training group supplemented with SLE. Glycemia levels were measured at 0, 15, 30, 60, and 120 min post-glucose administration (n = 6–8). *: difference between SPT and all other groups (*p* < 0.05). &: difference between C and SP groups (*p* < 0.05). (**B**): C (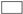), sedentary control group; SP (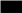), sedentary group supplemented with SLE; T (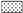), endurance training group; and SPT (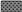), endurance training group supplemented with SLE. The total area under the curve (AUC) of A curves (expressed in arbitrary units, a.u.); bars represent the mean ± SEM for each group (n = 6–8), and letters (a > b) indicate statistically significant differences between groups (*p* < 0.05).

The glucose tolerance test (GTT) was conducted on the first day of the 7th week, 48 h after the last training session. Fifteen minutes after glucose injection, glycemia levels were significantly reduced in the SPT group compared to the other groups (Figure 3A).

Thirty minutes after injection, glycemia was significantly decreased in the SP group compared to the C one. In our conditions, an SLE effect was observed without any effect of endurance training. This is confirmed by the area under the curve (AUC) of the GTT, where groups receiving SLE showed a significant reduction compared to the C and T groups (Figure 3B).

### 3.5. Maximal Aerobic Speed

**Figure 4:**
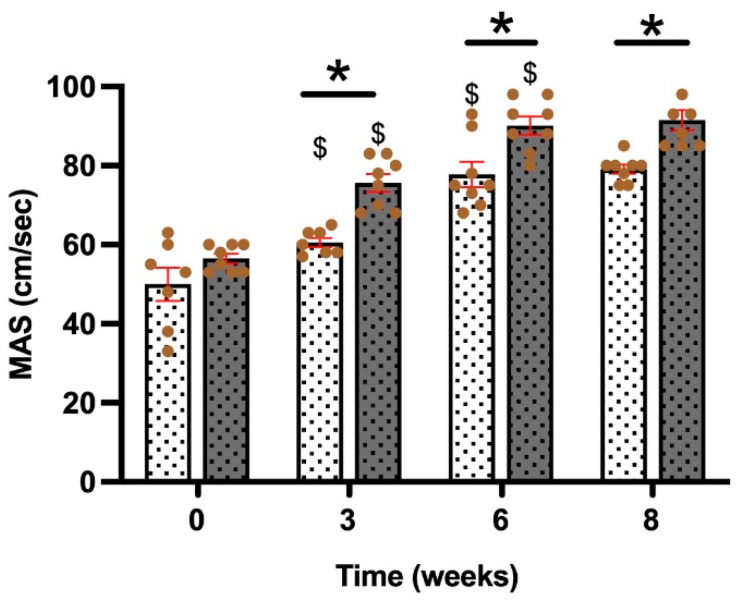
Maximal Aerobic Speed (MAS). The figure illustrates the changes in maximal aerobic speed (MAS, cm/s) over time (0, 3, 6, and 8 weeks) for two groups: T (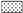), endurance training group; and SPT (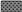), endurance training group supplemented with SLE. Bars represent the mean ± SEM for each group (n = 7–8), and brown dots represent individual values. *: indicates a significant difference between the T and SPT groups at the specified time point (*p* < 0.05). $: indicates a significant difference compared to the previous MAS measurement within the same group (*p* < 0.05).

Maximal aerobic speed (MAS) was measured as a marker of physical performance and assessed on a treadmill using exhaustion criteria [43]. During the 8 weeks of supplementation, MAS was measured four times (Figure 4). Before the first block, no difference was observed between each group, as an equality of performance. The following MAS test revealed an improvement for the SPT group compared to the T group (Figure 4). This difference persisted in the subsequent two measurements until the end of the 8 weeks. Additionally, in the 3rd and 6th weeks, both the T and SPT groups improved their own MAS records. Between the 6th and 8th weeks, a stagnation in MAS was observed (Figure 4).

### 3.6. Plasma Lipid Profile, Glucose, Insulin Concentrations, and Interleukin-6 Levels

The lipid profile was measured in plasma. No effect of SLE was observed, while during endurance training, triglyceride (TG), total cholesterol (T-Chol), HDL-Chol, and non-HDL-Chol levels decreased significantly (Table 4). The Atherogenic Index of Plasma (AIP) did not show any significant difference between the C and SP groups. However, during endurance training (C vs. T groups), AIP significantly decreased, with an even more marked reduction when SLE supplementation was added (T vs. SPT groups).

On the day of euthanasia, glycemia was measured, revealing an effect of SLE supplementation. A significant decrease in fasting blood glucose was observed in the SP and SPT groups compared to the C and T groups (Table 4). Additionally, plasma insulin levels showed a significant increase between the C and SP groups, as well as between the T and SPT groups, with the SPT group displaying a significantly higher insulin level compared to the SP group (Table 4). The level of IL-6 remained unchanged with SLE supplementation but increased significantly with endurance training.

### 3.7. Assessment of Lipid Peroxidation in Muscle: Malondialdehyde Content

**Figure 5:**
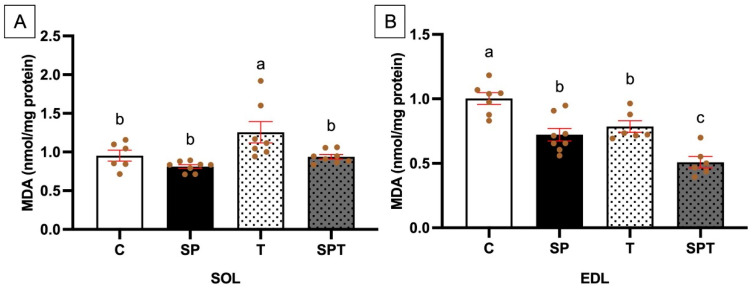
The figure illustrates MDA levels in SOL (**A**) and EDL (**B**) muscles. Groups: C (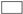), sedentary control group; SP (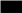), sedentary group supplemented with SLE; T (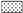), endurance training group; and SPT (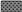), endurance training group supplemented with SLE. Bars represent the mean ± SEM for each group (n = 5–8), and brown dots represent individual values. Different letters (a > b > c) indicate statistically significant differences between groups (*p* < 0.05).

In the soleus muscle, endurance training significantly increased MDA content in the T group compared to the C group (Figure 5A). However, no significant effect of SLE supplementation was observed between the C and SP groups. When SLE was combined with endurance training (SPT), a significant decrease in MDA was observed compared to the T group (Figure 5A). In the extensor digitorum longus muscle, a significant decrease in MDA content was observed between the C and T groups, indicating a training effect (Figure 5B). SLE supplementation alone significantly decreased MDA content in the EDL, as reported in the C and SP groups (Figure 5B). Furthermore, when SLE was combined with exercise, MDA levels decreased even more, with a significant difference between the T and SPT groups (Figure 5B).

### 3.8. Gene Expression

#### 3.8.1. Gene Biomarker Modulation in Soleus and EDL Muscles by *Spirulina* Liquid Extract and Exercise Training

**Figure 6:**
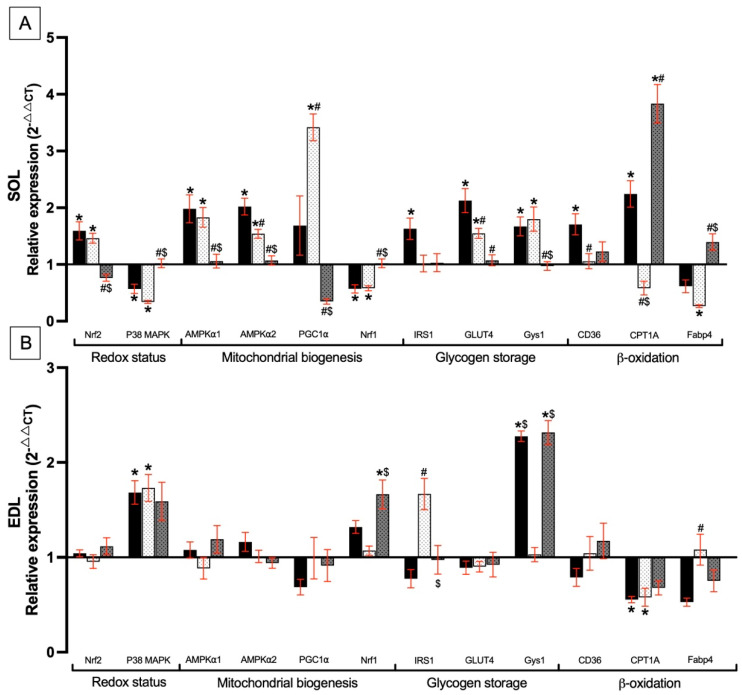
The effect of SLE (SP) and endurance training (T) on relative gene expression in SOL (**A**) and EDL (**B**) muscles. The figure represents relative gene expression normalized to the sedentary control group (not displayed). Bars represent the mean ± SEM for each group (n = 5–8): SP (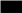), sedentary group supplemented with SLE; T (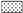), endurance training group; and SPT (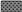), endurance training group supplemented with SLE. * indicates significant differences compared to the Control group. # indicates significant differences compared to the SP group. $ indicates significant differences compared to the T group (*p* < 0.05).

In the SOL muscle, gene expression was measured by RT-qPCR, with relative expression for the SP, T, and SPT groups normalized to C (Figure 6A). Regarding redox status in the SOL muscle, the Nrf2 gene was significantly upregulated, while the P38 MAPK gene was downregulated in both the SP and T groups. In contrast, in the SPT group, the expression of Nrf2 and P38 MAPK remained unchanged compared to the C group, but a significant difference was observed when compared to the SP and T groups. Concerning the mitochondrial biogenesis genes, the transcript abundance of AMPKα1 and AMPKα2 increased in both the SP and T groups. A similar upregulation pattern was observed for PGC1α in the T group but not in the SP group, whereas the expression level of Nrf1 decreased in both groups. In the SPT group, these genes remained unchanged compared to the C group, but with significant differences compared to the SP and T groups. For glycogen storage, IRS1 was significantly upregulated in the SP group but not in the T and SPT group. GLUT4 expression increased significantly in both the SP and T groups compared to C; however, no change was observed in the SPT group. Notably, GLUT4 expression was significantly downregulated in the SPT group compared to the SP group. The Gys1 gene was overexpressed in both SP and T groups compared to C, whereas no change was observed in the SPT group, which differed significantly from both the SP and T groups. Regarding β-oxidation metabolism, CD36 and CPT1A genes were upregulated in the SP group and CPT1A was overexpressed in the SPT group. Fabp4 transcript abundance was significantly reduced in both SP and T groups. Interestingly, in the SPT group, no change was observed compared to the C group; however, a significant increase was measured compared to the SP and T groups. In the EDL muscle, no difference in the expression level of Nrf2 was observed, whereas P38 MAPK was significantly upregulated in the SP, T, and SPT groups. However, the increase was not significant for the SPT group (*p* = 0.07) (Figure 6B). Mitochondria biogenesis genes, including AMPKα1, AMPKα2, and PGC1α, showed no significant variation in expression, while the Nrf1 gene was upregulated in the SPT group compared to the C and T group. Regarding glycogen storage, the expression of IRS1 significantly increased in the T group but not in the SP and SPT group. GLUT4 gene expression remained unchanged in all groups, whereas the Gys1 gene was overexpressed in the SP and SPT groups, showing a significant difference from the T group. For β-oxidation metabolism, CD36 transcript abundance exhibited no change in expression, and the CPT1A gene was downregulated in both SP, T, and SPT (*p* = 0.06) groups. The expression of Fabp4 decreased in the SP group (*p* = 0.06), but not in the T or SPT group.

#### 3.8.2. Effect of *Spirulina* Liquid Extract in Endurance Training Condition

**Figure 7:**
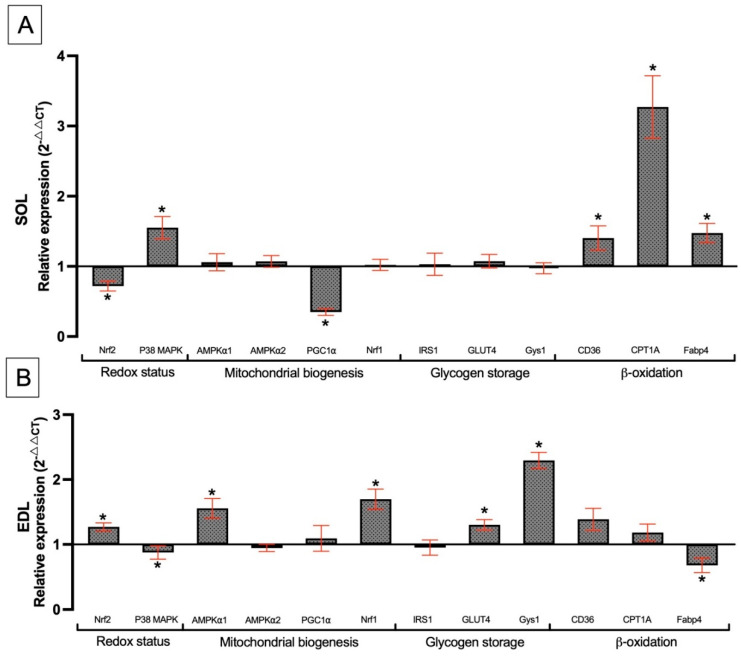
The effect of SLE on relative gene expression in SOL (**A**) and EDL (**B**) muscles in endurance training rats. The figure represents relative gene expression normalized to the training group (not displayed). Bars represent the mean ± SEM for each group (n = 5–8). * indicates significant differences compared to the training group (*p* < 0.05).

Gene expression was measured by RT-qPCR in both SOL and EDL muscles, with the relative expression for the SPT group normalized to the T group. In the SOL muscle, the Nrf2 gene was highly downregulated, while the P38 MAPK gene was upregulated (Figure 7A). Concerning the mitochondrial biogenesis genes, including AMPKα1, AMPKα2, and Nrf1, no change was shown in expression levels. However, the expression level of the PGC1α gene significantly decreased. In terms of glycogen storage, there was no significant variation in the expression of IRS1, GLUT4, or Gys1. Concerning β-oxidation metabolism, CD36, CPT1A, and Fabp4 were significantly upregulated. In the extensor digitorum longus muscle, the Nrf2 gene was upregulated, whereas P38 MAPK expression was downregulated (Figure 7B). Mitochondria biogenesis genes, including AMPKα1 and Nrf1, showed significant overexpression. However, no difference was observed in the expressions of AMPKα2 and PGC1α genes. Regarding the glycogen storage gene, only GLUT4 and Gys1 genes were upregulated. Finally, concerning genes of β-oxidation metabolism, only the Fabp4 gene expression was significantly downregulated.

## 4. Discussion

The monitoring of water and food intake showed that endurance activity is in agreement with previous studies explaining that endurance training is shown to decrease food intake in rats [44,45,46]. As an explanation, endurance training could mimic leptin signaling from the hypothalamus and decrease satiety [47]. In our conditions, SLE was not reported to have any effect on food intake. This could be due to phycocyanin, which has already been reported without any effect on this parameter [48,49]. Concerning drinking water, adding SLE led to an increase in water intake at weeks 1, 2, 6, and 7 in the SP and SPT groups compared to the C and T groups (Figure 1B). Taking into account the endurance training parameter, at the beginning of the study, water intake decreased in the T group compared to the C group (Figure 1B). However, from the 6th week onwards, no difference was observed between the C and T groups. Another study involving moderate exercise training in Wistar rats has also shown a decrease in water intake during 6 weeks [46].

It is known that endurance training reduces fat mass, leading to a decrease in BW compared to sedentary groups [50]. Thus, BW decrease could be related to a decrease in fat mass. Indeed, a decrease in adipose tissues (epididymal and peri-renal) was observed in the T and SPT groups, compared with the C and SP groups. SLE did not modify BW parameters in rats, which is consistent with findings from other studies in humans supplemented during 12 weeks [51] and in hamsters following a normocaloric diet supplemented for 2 weeks [29].

The weight of SOL and EDL muscles is normalized by tibia length and BW. The tibia length normalization is often more reliable because it is independent of BW and age. Therefore, using tibia length as a reference allows for an accurate measurement of tissue mass without interference from other parameters [52,53,54]. Thus, when referencing tibial length, no effect of training or SLE is observed. However, when normalized to body weight, it has been shown that the SOL mass increases with endurance training, while the EDL mass remains unchanged [55]. Here, our results converge, but in the presence of SLE, no differences were observed.

We are the first study that has measured a GTT in the presence of SLE supplementation. Thus, without any reference to the response to GTT, it can be proposed that our results are due to phycocyanin (PC). Indeed, it has already been reported that supplementation with 100 mg/kg of PC during 3 weeks reduced glycemia during this test in KKAy mice [56]. Thus, SLE could improve glucose response and thus reduce glucose intolerance during diabetes.

Daily aerobic training is known to increase aerobic capacity and the development of type I muscle fibers to improve cardiovascular and respiratory efficiencies, and its better use of energy reserves such as lipids [57]. In the study by Pengam et al., the differences between moderate-intensity continuous training (MICT) and high-intensity interval training (HIIT) have been evaluated. It has been demonstrated that HIIT improved MAS performance over 6 weeks. In our study, we achieved a similar effect to the HIIT protocol by combining MICT and HIIT training [58]. Indeed, during our protocol, sprints were added to endurance activity. To our knowledge, it is the first time reported that SLE supplementation significantly increased MAS performance. However, a previous study reported the effect of *Spirulina* supplementation during 4 weeks in humans [59]. It has been demonstrated that time to fatigue after a 2 h run was significantly longer with *Spirulina* supplementation, which is similar to the MAS measurement in our present study. A decrease in TBARS content was observed, proposing that *Spirulina* enhanced lipid peroxidation, thereby improving performance. It must be noticed that *Spirulina* biomass contains numerous different molecules that could contribute to this improvement. In our case, we propose that SLE, with phycocyanin extracted from *Spirulina*, could act as an antioxidant, enhancing lipid peroxidation and improving performance, as observed in the MAS measurements.

The plasma T-Chol is dependent on the availability of fatty acids (FA) in the liver [60]. During exercise, the TG and T-Chol concentrations decreased, likely due to an increase in fatty acid oxidation by skeletal muscle [61]. No significant effect of SLE was observed on the TG and T-Chol concentrations, which is consistent with the study of Koite et al. in humans that also did not find any effect [51]. However, the study by Kasbi-Chadli et al. in a hamster model showed a decrease in TG content in plasma, but not in T-Chol [29]. In the present study, plasma HDL-Chol levels decreased with endurance training, while moderate exercise over 4 weeks in Wistar rats was shown to significantly increase HDL-Chol, which could be correlated with LXRα gene expression in the liver [62]. Nevertheless, according to our results, SLE would be able to limit the decrease in HDL-Chol during endurance training. As in previous studies, it was reported that SLE supplementation did not affect plasma HDL-Chol [29,51]. It can be proposed that the combination of SLE with endurance training for 8 weeks is responsible for the decrease in HDL-Chol. The non-HDL-Chol content likely includes other forms of cholesterol, such as low-density lipoprotein (LDL). It is accepted that the LDL-Chol level decreases during exercise [62], and this was observed in the present study (in the C group compared to the T group). The AIP is an effective marker of cardiovascular disease risk [40]. We observed a decrease in lipid levels during exercise (in the C group compared to the T group), with a more pronounced decrease when combined with SLE (T vs. SPT groups). While no difference was observed between the C and SP groups, this could be due to the observed trend between the T and SPT groups, where TG content decreased while HDL-Chol levels increased in the SPT group. According to previous studies related to the effect of *Spirulina* on AIP decrease, we can explain that our results are due to phycocyanin present in SLE [63]. Thus, our results show a cumulative effect of SLE and endurance training to prevent cardiovascular diseases.

On the other hand, the decrease in fasting blood glucose measured in the SLE groups is likely due to the higher insulin levels in plasma, as has already been proposed about the role of phycocyanin in the decrease in fasting blood glucose and the increase in plasma insulin levels [64]. Other studies proposed that phycocyanin (PC) can improve pancreatic beta-cell function by activating the phosphatidylinositol 3-kinase/protein kinase B (PI3K/Akt) pathways, thus protecting methylglyoxal, a glycation agent linked to cell damage in diabetes [65]. Thus, PC could have protective effects against diabetes. Our data showed that SLE increased plasma insulin and that insulin sensitivity was improved with better glycemic regulation, as observed with previous GTT results (Figure 3A,B). This could improve physical capacity, as recently described, with PC supplementation [66]. Moreover, recent studies have demonstrated that increased insulin sensitivity was associated with better physical performance, due to an efficient use of glucose as an energy source. This parameter can also positively influence post-exercise recovery, reducing the risk of fatigue and injury [67]. Thus, in the present study, the improvement in MAS with SP could be explained by an increased insulin sensitivity.

We have measured that IL-6 increased with endurance training. This may be due to muscle contractions, which release IL-6 into the bloodstream. The increased IL-6 level stimulated lipolysis in adipose tissue, promoted anti-inflammatory macrophage activity, and increased insulin levels [68]. Moreover, circulating IL-6 plays a role in glucose oxidation and enhances recovery after exercise, including the rebuilding of contractile tissue [69].

The MDA is a marker of lipid peroxidation and is used to predict oxidative stress levels [70]. The interest in studying the SOL muscle is due to its high composition of slow-twitch fibers, while the EDL is composed mainly of fast-twitch fibers [71,72]. Both muscles are frequently used in running training studies [73]. It has been reported in Wistar-Kyoto rats that endurance training increases MDA levels in the SOL muscle due to the relationship between aerobic exercise, increased mitochondrial electron transport, and the resulting superoxide radical leakage [74,75]. This was also observed in the present study. The SLE supplementation in the SPT group significantly decreased MDA content in the SOL muscle during training, indicating a protective effect against oxidative stress. A recent study demonstrated that PC supplementation increased antioxidant enzyme activity and decreased oxidative stress in the quadriceps muscles of Sprague Dawley rats [66]. During this study, the authors used the swim test performance considered similar to our MAS measurements. They observed the beneficial effects of PC. In the present study, the significant decrease in MDA observed in the SPT group in the SOL muscle could similarly contribute to improved performance—as seen in the MAS measurements—and this could be explained by the content of PC in the SLE.

In the EDL muscle, it has been suggested that MDA levels decrease during 12 weeks of endurance exercise because glutathione peroxidase may be overexpressed, reducing oxidative stress and preventing lipid peroxidation in fast-twitch fibers [76]. Excessive oxidative stress can impair muscle performance by reducing calcium sensitivity and damaging muscle proteins [77]. The decrease in MDA content observed in our training groups suggested an adaptive response to endurance training, which helps maintain muscle function [13]. In the SP group, MDA content also decreased significantly, suggesting that SLE may have a similar antioxidant effect to exercise. The combined effect of SLE and exercise further decreased MDA content in the EDL muscle, which may have contributed to the improved MAS performance. These results in both the SOL and EDL muscles suggest that SLE has an antioxidant effect when combined with exercise.

### 4.1. Redox Status Genes in the Soleus Muscle

Endurance training is known to produce oxidative stress due to significant mitochondrial demand [78]. This oxidative stress can lead to low-level systemic inflammation, whereas exercise has been shown to exert an anti-inflammatory effect [79]. The Nrf2 factor negatively regulates inflammation during exercise and regulates antioxidant enzymes [80]. In this work, it has been proposed that Nrf2’s protective role is crucial for maintaining muscle performance and mitigating the long-term effects of exercise-induced stress (Figure 6A). Consequently, the increase in Nrf2 gene expression observed during endurance training in the SOL could counteract the elevated production of reactive oxygen species (ROS) generated by mitochondrial oxidative capacity. Furthermore, in the SP group, Nrf2 gene expression increased as it did with exercise. This result was observed in other studies that supplemented with PC, suggesting that PC acts as an activator of Nrf2 pathways [26,66]. Interestingly, the combination group (SPT) did not show any difference compared to group C. This could be explained by an improved recovery with better regulation of oxidative status due to the combination of SLE and endurance training. The P38 MAPK is a redox-sensitive protein, as described by Vichaiwong et al., and its gene expression decreased in both groups in this study (SP and T groups) [81]. This effect is likely due to the overexpression of the Nrf2 gene, which reduces ROS levels, as demonstrated in liver studies [82]. However, in the SPT group, P38 MAPK expression remained unchanged, which is consistent with the fact that there are low levels of oxidative molecules, insufficient to induce Nrf2 expression. Thus, these results for the SPT group can be related to the obtained MDA content, which decreased in the SPT group compared to the T group (Figure 5A). Finally, these improvements in oxidative status could be related to better MAS measurements, as previously observed.

### 4.2. Mitochondrial Biogenesis Genes in the Soleus Muscle

AMPK exists in two isoforms: AMPKα1 and AMPKα2, and both play a role in muscle mass pathways, muscle regeneration, and energy management during and after a physical exercise [83,84]. AMPK activation following endurance training indicated muscle adaptation with glycogen synthesis or storage [83] and with mitochondrial biogenesis [80]. This is supported by the significant upregulation of GLUT4 and PGC1α gene expressions in the T group. However, the AMPKα1 and AMPKα2 genes were upregulated in the SLE group without endurance training. Thus, the SLE could be able to stimulate this AMPK pathway and have a positive effect on AMPK activation as endurance training. PGC1α is considered a key regulator of mitochondrial production and function in muscle [85,86]. Another important regulator is Nrf1, playing a role in mitochondrial transcriptional activity [87]. Both can negatively regulate each other to maintain mitochondrial homeostasis [87]. In our study, the Nrf1 expression downregulation in T and SPT groups could be explained by this regulatory interaction with PGC1α [87]. Results reported in Figure 6A showed that SLE induced a gene regulation related to mitochondrial biogenesis similar to during endurance training (T). Interestingly, when SLE was combined with endurance training (SPT group), the expression of AMPKα1, AMPKα2, PGC1α, and Nrf1 genes remained unchanged. This is likely due to the low levels of oxidative molecules and the overall muscle recovery. Indeed, other studies on human muscle and acute exercise have reported that mRNA levels of these genes increase after exercise and return to basal levels during recovery. [88,89]. These data suggest that physical capacity could be improved with SLE supplementation without the need for training, as we observed similar effects to training alone. The combination of SLE supplementation and training seems to enhance recovery speed and muscle adaptation in response to our training protocol. Finally, this could explain the improvement in the MAS test observed in the SPT group.

### 4.3. Glycogen Metabolism and Storage Genes in the Soleus Muscle

The SP group exhibited a significant IRS1 upregulation, likely due to elevated circulating insulin levels, as it has already been reported (Table 4). GLUT4 and Gys1 gene expressions upregulated with training (T group), as already demonstrated in human studies [90]. Additionally, research on Gys1 knockout mice has observed an 85% reduction in Gys1 mRNA and a 70% reduction in glycogen protein content in the SOL due to the inhibition of the Gys1 gene [91]. Thus, it can be considered that the upregulation of GLUT4 and Gys1 genes in response to training (Figure 6A) could reflect a restoration of glycogen storage for subsequent sessions. In the SP group, the same upregulation was noticed, suggesting a potential protective role against insulin resistance as it has already been reported [92]. Thus, we hypothesize that SLE could prevent insulin resistance and enhance muscle performance by improving glycogen storage regulation. Interestingly, the combination of both conditions (SLE and endurance training) did not induce any change in the relative expression of glycogen-related genes observed compared to the control group. These results could also explain an overall adaptation of glycogen metabolism and storage following exercise.

### 4.4. Fatty Acid Metabolism Genes in the Soleus Muscle

Regarding β-oxidation, the SP and SPT groups exhibited significant increases in CPT1A gene expression (Figure 6A). Increased fatty acid oxidation capacity through CPT1 has been shown to contribute to muscle plasticity and remodeling [93]. This could also contribute to the enhancement of physical performance observed in the SPT group following endurance training. In our study, Fabp4 gene expression was downregulated in the T group. This gene is recognized as a marker of mature adipocytes in muscle and is implicated in intramuscular adipose tissue (IMAT) development leading to muscle atrophy and strength loss [94,95]. Despite an increase in the expression of fatty acid transport genes like the CD36 gene in the SP group, the elevated expression of CPT1A in this group may lead to mitochondrial uncoupling as previously reported [96].

### 4.5. Redox Status Genes in the EDL Muscle

The level of Nrf2 expression did not change in the SP, T, and SPT groups (Figure 6B). This may be due to the low mitochondrial content in this muscle, which may not require the same level of regulation for counteracting ROS overproduction [97]. A high expression of the P38 MAPK gene was observed in the SP, T, and SPT (*p* = 0.07) groups, with an upregulation of Gys1 expression in the latter. In the EDL muscle, the P38 MAPK gene cannot be considered a marker of oxidative stress as it is in the SOL muscle, while it is a marker for glucose uptake independently of insulin regulation [98,99]. This suggested that SLE may enhance glycogen storage independently of insulin in the EDL muscle, similar to the effect of exercise training.

### 4.6. Mitochondrial Biogenesis Pathways Genes in the EDL Muscle

Regarding gene markers of mitochondrial biogenesis, no effect was observed in the T group (Figure 6B). This is probably due to the type of muscle fibers mainly composed of type II fast fibers in which glycogen metabolism is dominant [100]. However, in the SPT group, we observed an overexpression of the Nrf1 gene. This could be explained by the switch of glycolytic fibers to oxidative fibers, given that Nrf1 plays a role in mitochondrial biogenesis [80,101]. Thus, SLE supplementation combined with exercise training could improve endurance performance through the remodeling of fiber energy metabolism. Moreover, SLE alone (SP group) tended to increase Nrf1 mRNA, while the endurance training group (T) without SLE showed no change in expression, suggesting that SLE may act as a regulator of fiber-type switching.

### 4.7. Fatty Acids and Glycogen Metabolism Genes in the EDL Muscle

Markers for fatty acid transport remained unchanged in the SP, T, and SPT groups, while CPT1A gene expression decreased (Figure 6B). However, Gys1 mRNA was upregulated in both SLE groups. According to the Randle cycle, elevated glycogen levels can negatively regulate fatty acid oxidation [102]. Thus, in our study, this mechanism could be observed in both SLE groups. On the other hand, CPT1A gene expression also decreased in the training group, with an overexpression of IRS1 mRNA, which may also reduce the regulation of fatty acid metabolism. Related to the decreased Fabp4 gene expression in the SP group (*p* = 0.06) (Figure 6B), SLE consumption could prevent the development of IMAT as proposed for the SOL muscle.

### 4.8. Genes Associated with the Effects of SLE Supplementation Under Endurance Training Conditions in the Soleus Muscle

We aimed to observe the differences between the T and SPT groups through various relative gene expressions. These results would explain how MAS performance increased in the SPT group compared to the T group. Here, the Nrf2 gene expression was downregulated, whereas the data reported in Figure 6A showed that this expression was upregulated in the SP group. Thus, we expected that SLE, in combination with endurance training, would upregulate the expression of these genes. Conversely, the P38 MAPK gene expression increased, suggesting a higher presence of ROS in the SPT group compared to the T group (Figure 7A). In addition, PGC1α gene expression significantly decreased, which may correspond to a reduction in mitochondrial adaptation [86]. The difference in levels of expression between Nrf2, P38 MAPK, and PGC1α genes, suggests that SLE, when combined with endurance training, may alter mitochondrial adaptation. This observation could be similar to the effects of antioxidant supplementation with vitamins C or E [17]. Thus, SLE may act as a primary antioxidant in the SOL muscle during endurance conditions. As an explanation, the decrease in the expression of PGC1α could be balanced by the Nrf2 gene, which decreased and generated an increase in ROS concentration as indicated by the high expression of P38 MAPK [103]. No difference in glycogen pathway storage was observed between the different groups; however, there was a significant upregulation in the expression of β-oxidation genes such as CD36 and CPT1A. This improvement was also observed with SLE supplementation (Figure 6A). The effect is enhanced by the combination of SLE supplementation and endurance training (Figure 7A). Physical exercise increases the transport of fatty acids into muscle cells, and CD36 plays a role in fatty acids substrate selection to enhance exercise performance [104]. In Figure 6A, it has been reported that expression of CD36 was unchanged in the T group. However, with SLE supplementation and endurance training (SPT), an upregulation of this gene expression was observed (Figure 7A). This could lead to an improvement in fatty acid regulation with a better efficiency for fat oxidation. Moreover, the CPT1A gene was overexpressed, as previously demonstrated in rats, where CPT1A expression is regulated by thyroid and pancreatic hormones, as well as long-chain fatty acids [105]. It has been reported that an increase in CPT1 improves fatty acid oxidation, leading to better physical performance [106]. Previously, we have reported that CPT1A was upregulated in the SP group (Figure 6A), while in the T group, CPT1A was downregulated. We also observed this upper expression in the SPT group compared with the T group (Figure 7A). Thus, it can be hypothesized that SLE had a stimulating effect on the CPT1A gene pathway. These results could explain the improvements observed in the incremental test to exhaustion (MAS) in the SPT group compared to the T group. Moreover, SLE supplementation could lead to better regulation of fat utilization with a protective role during metabolic syndrome such as obesity or diabetes. Additionally, Fabp4 expression was upregulated in the SPT group, likely due to fatty acid storage for use as an energy substrate [107]. This explanation can be related to the downregulated Fabp4 expression observed with SLE supplementation (Figure 6A).

### 4.9. Genes Associated with the Effects of SLE Supplementation Under Endurance Training Conditions in the EDL Muscle

We have reported that an increase in plasma IL-6 levels induced by endurance training may be related to the increase in GLUT4 and Gys1 expression genes [108]. Previous results in the EDL muscle showed that Gys1 expression was upregulated with SLE supplementation and without endurance training (Figure 6B). Here, we also observed an increase in Gys1 expression with both endurance training and SLE supplementation (Figure 7B). This could be a critical factor explaining the increase in physical capacity during MAS tests [109]. Additionally, the overexpression of AMPKα1 and Nrf1 genes could contribute to improved energy metabolism adaptations, leading to better performance [110,111]. Contrary to the result obtained in the SOL muscle, Nrf2 gene expression increased with SLE and endurance training in the EDL muscle, while P38 MAPK gene expression decreased. As previously suggested, it can be confirmed that SLE has different effects according to muscle fiber type.

## 5. Conclusions

This study conducted on male Wistar rats supports the initial hypothesis that SLE supplementation can enhance performance and promote molecular adaptations in the SOL and EDL muscles during endurance training. The antioxidant effects of phycocyanin in SLE appear to improve exercise performance, likely through remodeling in energy metabolism. However, the complex interactions between SLE supplementation and training-induced adaptations, particularly regarding mitochondrial biogenesis, justify further investigations to fully understand the implications for athletic performance and muscle health. Although conducted on male Wistar rats, our results highlight the potential of SLE as a functional food supplement that could be used for athletes with a view to training outcome optimization. By modulating oxidative stress levels, SLE could serve as an effective strategy for enhancing recovery and performance. Future research should explore the long-term effects of SLE supplementation, and its impact on different exercise modalities.

## Figures and Tables

**Table 1:** Training protocol conducted for 8 weeks with 1 week of adaptation.

	Weeks	Running Time (min)	Speed (cm/s)	MAS Measurement	Sprints
**Adaptation**	1	10	11–16–21	X	
**Block 1**	2	25	60% average MAS		
	3	30	70% average MAS		
	4	30	80% average MAS	X	
**Block 2**	5	60	60% average MAS		30 s at 70% of the average MAS every 10 min
	6	60	70% average MAS		30 s at 80% of the average MAS every 10 min
	7	60	80% average MAS	X	30 s at 90% of the average MAS every 10 min
**Block 3**	8	60–70	60% average MAS		1 min at 80% of the average MAS every 13 min
	9	60	70% average MAS	X	1 min at 90% of the average MAS every 13 min

**Table 2:** Primers sequences for the real-time PCR reaction.

Genes	Abbreviations	Forward Primers	Reverse Primers
**Redox status**			
**P38 Mitogen-Activated Protein Kinases**	P38MAPK	TGCCAGGAATGGAGCCACAT	CATGGAGAAAACTGCCGCCC
**Nuclear respiratory factor 2**	Nrf2	ATGCCTTCCTCTGCTGCCAT	TCGGCTGGGACTTGTGTTCA
**Β-oxidation**			
**Carnitine Palmitoyltransférase 1A**	CPT1A	AGCTGGATCTGGGAAACGGG	TCGATCAATGTGGGGAGGCC
**Fatty acid binding protein 4**	Fabp4	GCTGTACTGTCTGGGCCTCA	ATGAGAGGGTGGAGGTGCAG
**Cluster of Differentiation 36**	CD36	ACTAAGCTGTTTGTGTGCCCC	ATCATCGAGTGGTGCTACTGGT
**Glycogen storage**			
**Glucose Transporter 4**	GLUT4	TTGACCAGATCTCGGCCACC	AGTGCTGCGAGGAAAGGAGG
**Glycogen synthase 1**	Gys1	ACCCCATTCCCTGCCTGTTC	GCGATCCAAGAAAGGCACGG
**Insulin Receptor Substrate 1**	IRS1	GCACCTCTCATTCAGCCCCT	CATGCAGCGTTTGTGGACCA
**Mitochondrial biogenesis pathway**			
**Protein kinase AMP-activated catalytic subunit alpha 1**	AMPKα1	TGGCTTCAGTCACCATCACCA	AGCTCCCACAACTCAGGCTC
**Protein kinase AMP-activated catalytic subunit alpha 2**	AMPKα2	AGGTGGTGGAGCAGAGGTCT	GGGGAAGCGGAGGACAAAGT
**PPARG Coactivator 1 alpha**	PGC1α	TTCCAGAAGCTCCAGTGCCC	CCTGCTCAGCCATGCCTACT
**Nuclear respiratory factor 1**	Nrf1	CGCTGGTGTCCCTGGATCTT	CGAGTTAGGGTGTGGCAGGT
**Housekeeping gene**			
**Tubulin alpha 1**	Tubα	CCACTTCCCTCTGGCCACTT	GGGACCACATCACCACGGTA

**Table 3:** Effects of SLE and training on physical and nutritional parameters in rats.

Parameters	C	SP	T	SPT	n
**Characteristics**					
**Bodyweight (g)**	454 ± 9 ^a^	466 ± 10 ^a^	421 ± 5 ^b^	405 ± 10 ^b^	5–7
**Soleus (mg/100g BW)**	44.5 ± 1.3 ^b^	45.1 ± 1.5 ^b^	51.3 ± 1.4 ^a^	48.3 ± 1.8 ^ab^	7–8
**EDL (mg/100g BW)**	43.3 ± 0.9 ^ab^	40.7 ± 1.9 ^b^	43.7± 1.8 ^ab^	47.4 ± 0.7 ^a^	8
**Tibial length (cm)**	4.10 ± 0.01 ^a^	4.00 ± 0.05 ^a^	4.11 ± 0.02 ^a^	3.96 ± 0.04 ^a^	8
**Soleus (mg/cm TL)**	48.0 ± 1.1 ^a^	53.8 ± 2.5 ^a^	52.6 ± 1.9 ^a^	50.2 ± 2.4 ^a^	8
**EDL (mg/cm TL)**	46.8 ± 1.1 ^a^	49.8 ± 2.7 ^a^	45.6 ± 1.8 ^a^	48.7 ± 1.3 ^a^	8
**Nutritional parameters**					
**Food intake (g/day/100g BW)**	7.5 ± 0.2 ^a^	7.6 ± 0.2 ^a^	7.1 ± 0.2 ^a^	7.3 ± 0.2 ^a^	8
**Water intake (mL/day/100g BW)**	8.8 ± 0.2 ^bc^	9.7 ± 0.3 ^ab^	8.4 ± 0.2 ^c^	10.3 ± 0.3 ^a^	8
**Food intake/weight gain to W1 at W4** **(pearson correlation)**	r = −0.4074	r = −0.2302	r = 0.0075	r = 0.1839	8
	*p* = 0.0206	*p* = 0.0695	*p* = 0.9673	*p* = 0.1490	
**Food intake/weight gain to W4 at W8** **(pearson correlation)**	r = 0.5340	r = 0.1219	r = 0.0519	r = 0.6341	8
	*p* = 0.0016	*p* = 0.3414	*p* = 0.7777	*p* < 0.0001	
**Food intake/weight gain** **(pearson correlation)**	r = −0.0523	r = 0.0742	r = −0.2176	r = 0.2285	8
	*p* = 0.6814	*p* = 0.5601	*p* = 0.0882	*p* = 0.0694	

The table presents mean values ± SEM of various physical and nutritional parameters measured in the four groups of rats: C, sedentary control group; SP, sedentary group supplemented with SLE; T, endurance training group; and SPT, endurance training group supplemented with SLE. The sample size for each parameter is indicated in the n column. Physical characteristics include body weight, soleus, and extensor digitorum longus (EDL) muscle weight relative to 100 g of bodyweight or tibial length. Nutritional parameters include daily food and water intake. Pearson correlation coefficients (r) and associated *p*-values are provided to indicate the strength and significance of the relationships between food intake and weight gain across various time points. Groups with different superscript letters (a > b > c) indicate statistically significant differences between them (*p* < 0.05).

**Table 4:** Plasma parameters.

Plasma Parameters	C	SP	T	SPT	n
**TG (mmol/L)**	2.69 ± 0.39 ^a^	2.34 ± 0.21 ^ab^	1.60 ± 0.21 ^bc^	1.11 ± 0.10 ^c^	6–8
**T-Chol (mmol/L)**	1.62 ± 0.09 ^ab^	1.76 ± 0.12 ^a^	1.26 ± 0.07 ^c^	1.38 ± 0.06 ^bc^	5–7
**HDL-Chol (mmol/L)**	0.48 ± 0.03 ^a^	0.52 ± 0.03 ^a^	0.35 ± 0.03 ^b^	0.43 ± 0.01 ^ab^	5–7
**Non HDL-Chol (mmol/L)**	1.09 ± 0.08 ^ab^	1.12 ± 0.06 ^a^	0.83 ± 0.04 ^c^	0.85 ± 0.05 ^bc^	6–7
**AIP**	0.80 ± 0.05 ^a^	0.80 ± 0.01 ^a^	0.63 ± 0.04 ^b^	0.43 ± 0.03 ^c^	5–7
**Glycemia (mg/dL)**	60.8 ± 1.3 ^a^	55.2 ± 1.6 ^b^	64.8 ± 2.3 ^a^	50.3 ± 1.0 ^b^	5–7
**Insulin (µUI/mL)**	34.9 ± 3.3 ^c^	53.1 ± 3.9 ^b^	46.3 ± 5.7 ^bc^	82.3 ± 3.5 ^a^	5–6
**IL-6 (pg/mL)**	95.67 ± 9 ^b^	102 ± 15 ^b^	160 ± 18 ^a^	158 ± 16 ^a^	5–6

Biochemical plasma parameters of rats at the end of the study period following different conditions: C, sedentary control group; SP, sedentary group supplemented with SLE; T, endurance training group; and SPT, endurance training group supplemented with SLE. The values are expressed as mean ± SEM. The number of animals per group (n) is indicated in the last column. TG (mmol/L): triglycerides; T-Chol (mmol/L): total cholesterol; HDL-Chol (mmol/L): high-density lipoprotein cholesterol; non-HDL-Chol (mmol/L): non-high-density lipoprotein cholesterol; AIP: atherogenic index of plasma; Glycemia (mg/dL): blood glucose; Insulin (µUI/L): plasma insulin; IL-6 (pg/mL): interleukin-6. Groups with different superscript letters (a > b > c) indicate statistically significant differences between them (*p* < 0.05).

## Data Availability

The original data presented in the study are included in the article. Further inquiries can be directed to the corresponding author.
